# Learning to sense three-dimensional shape deformation of a single multimode fiber

**DOI:** 10.1038/s41598-022-15781-8

**Published:** 2022-07-25

**Authors:** Xuechun Wang, Yufei Wang, Ketao Zhang, Kaspar Althoefer, Lei Su

**Affiliations:** School of Engineering and Materials Science, Queen Mary University of London, London, E1 4NS UK

**Keywords:** Electrical and electronic engineering, Optical sensors

## Abstract

Optical fiber bending, deformation or shape sensing are important measurement technologies and have been widely deployed in various applications including healthcare, structural monitoring and robotics. However, existing optical fiber bending sensors require complex sensor structures and interrogation systems. Here, inspired by the recent renewed interest in information-rich multimode optical fibers, we show that the multimode fiber (MMF) output speckles contain the three-dimensional (3D) geometric shape information of the MMF itself. We demonstrate proof-of-concept 3D multi-point deformation sensing via a single multimode fiber by using *k*-nearest neighbor (KNN) machine learning algorithm, and achieve a classification accuracy close to 100%. Our results show that a single MMF based deformation sensor is excellent in terms of system simplicity, resolution and sensitivity, and can be a promising candidate in deformation monitoring or shape-sensing applications.

## Introduction

Complex deformation, bending or shape sensing has received growing attention, due to their booming applications in interactive sensing devices^1–4^ and soft robots^5–8^. Optical fiber sensors provide an efficient and competitive sensing solution, owning to their unique properties, including light-weight, low cost, freedom from electromagnetic interference, and resistance to harsh environment and chemicals^9–11^. Among optical fiber bending sensors, fiber Bragg gratings (FBGs)^12–14^, long period fiber gratings (LPFGs)^15,16^ and specially designed optical fibers^17,18^ are popular choices owing to their robustness. For example, the fiber sensor presented in Ref.^19^ exhibits high sensitivity (up to 1270 pm/°) and can be used to monitor small bending angles (2°). In Ref.^20^, a two-dimensional bending sensor based on long period gratings (LPGs) fabricated in three-core fibers were demonstrated. Furthermore, FBGs were fabricated in a six-core fiber to achieve a spatial resolution down to 40 µm^21^. While these existing fiber-optic sensors have demonstrated good sensitivity and resolution, the application of these previous optical-fiber-shape sensors are limited by the complex fabrication process and expensive detection systems. The fabrication of high-accuracy FBGs and good-performance multicore fibers is complicated. The signal processing and interrogation systems involved are bulky and high cost, which especially limits their application in wearable and portable devices. Furthermore, the spatial resolution of fiber-grating-based bending sensors is restricted by the minimal length of individual gratings that need to be incorporated to measure each deformation point. Therefore, a deformation sensor consisting of only standard and low-cost fibers would be ideal.

Multimode fibers (MMFs) are high-capacity information channels^22–24^. The MMF output speckles contain rich information and have been used in applications such as spectrometers^25^, optical trapping^26^ and imaging^27^. Very recently, utilizing MMF output speckles, researchers employed deep learning in input-pattern reconstructions, including MINIST characters^28,29^, natural scenes^30^ and even randomly distributed spatial information^31^. Here, we hypothesize that the MMF speckles possess the information that relates to the complex deformation along the fiber itself. In this work. We attempt to use machine learning to interpret the complex deformation of the fiber with output speckles. In fact, speckle-based MMF bending classification was recently reported^32–34^ on one-point low dimensional to classify such MMF bending. In Ref.^34^, a spring needle was used to press the fiber to achieve bending at one point, where the freedom of deformation is relatively low as both ends of the fiber were fixed. In this work, we propose the following to experimentally study speckle-based 3D multi-point deformation classification through a single MMF.

## Experiment and results

### One-point deformation experiment

**Figure 1:**
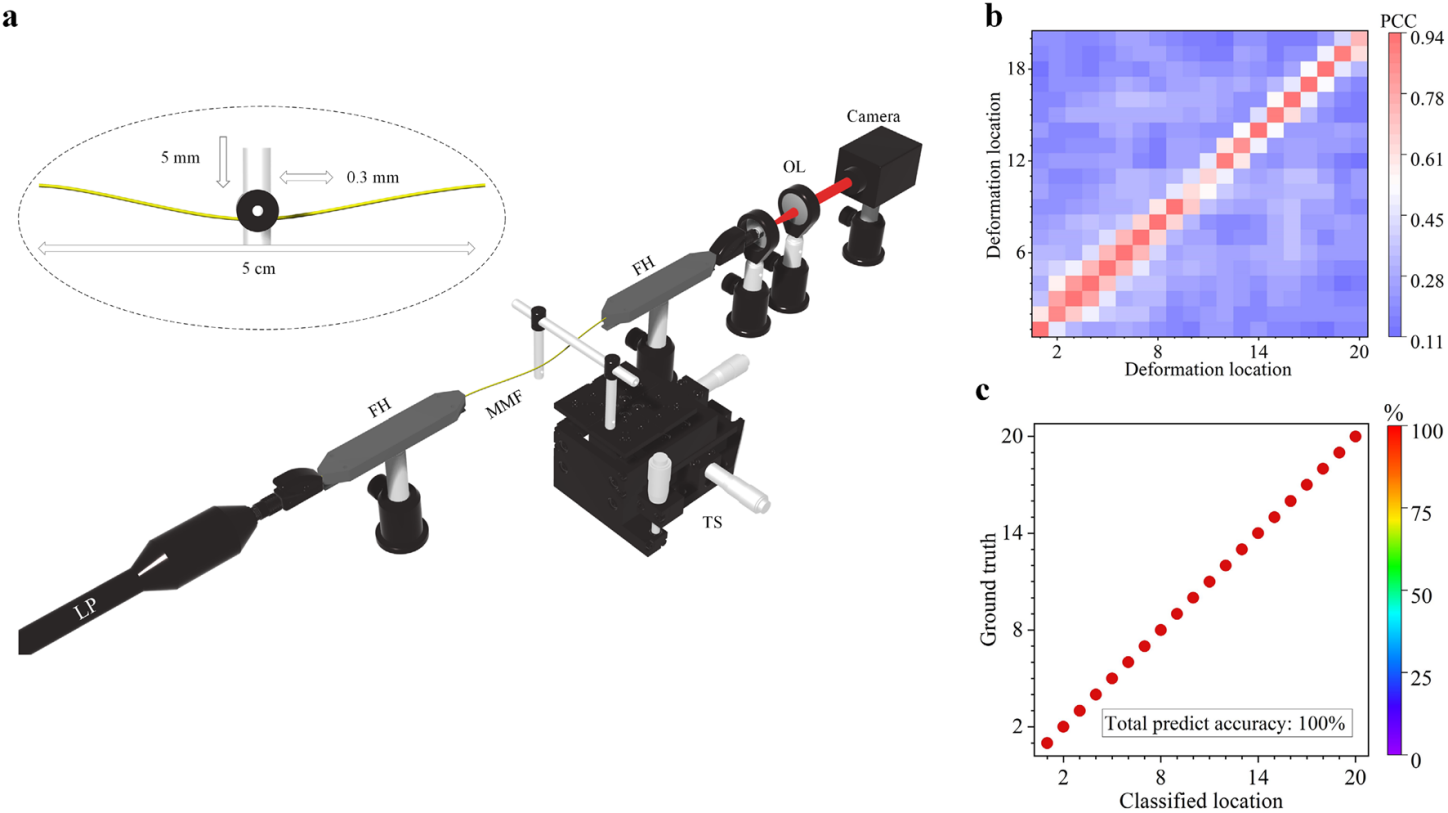
(**a**) Experiment setup for one-point deformation classification. (**b**) Average PCC for one-point deformation (the average PCC between the speckles collected in the first measurement and subsequently collected speckles in the rest 39 measurements). (**c**) Normalized confusion matrices for KNN classification results with one-point deformation experiment. KNN is trained by using only 50% dataset. *LP* Laser pointer, *FH* fiber holder, *TS* translation stage, *OL* objective lens.

Firstly, we studied the bending-location classification along the MMF axial direction using MMF speckles. As shown in Fig. 1 (see “Methods” section for details), we used a 11 cm long MMF and fixed a 5 cm section of this MMF between two translation stages as the deformation region. A 6 mm diameter steel rod controlled by a translation stage was used to induce one-point deformation within the 5 cm bending region. The fiber was carefully aligned to avoid twisting during deformation and was also stabilized so that the rest of the MMF (except for the bending point) remained undisturbed. The deformation was then formed by moving the steel rod perpendicular to the fiber axial direction for 5 mm to bend the fiber at one point. The camera was used to acquire an output speckle image 5 s after the deformation was applied, leaving enough time for the MMF and the speckle to stabilize. The steel rod was then translated 0.3 mm along the axial direction of the fixed MMF and the speckle acquisition was repeated. Having acquired output speckles at 20 different fiber bending points, we started a new loop by moving the steel rod backwards along the MMF axial direction for another repeating measurements. This process was repeated until we collected 40 speckles for each deformation location of the MMF. Finally, within 2 h we collected in total 800 speckle images, which were subsequently used for the deformation classification.

Figure 1b shows the average Pearson correlation coefficients (PCC) between the speckles collected in the first measurement and speckles collected in the rest measurements. It can be seen in Fig. 1b that, although the average PCC between neighboring bending locations are high between 0.6 and 0.7. The correlation coefficient decreased to 0.2 after a few times of deformation. Bending locations can still be accurately classified because the PCC between speckles collected at the same bending location is higher. The classification results using *k*-nearest neighbors algorithm (KNN) are shown in Fig. 1c. The classification accuracy is defined as the percentage of the correctly predicted speckles within test datasets (50% of the total datasets). Here we did not randomly select the training and testing datasets from the 800 datasets. Instead, to show the stability of the MMF-speckle based bending sensor, speckles recorded during the first half of the repeating speckle collections (i.e. the first 20 repeating measurements in the first hour of the 2-h measurements) were used as the training data and the rest were used as the test data. In Fig. 1c, the numbers of the diagonal elements show the percentage of correctly classified speckles, where a 100% classification accuracy is achieved for each bending location.

**Figure 2:**
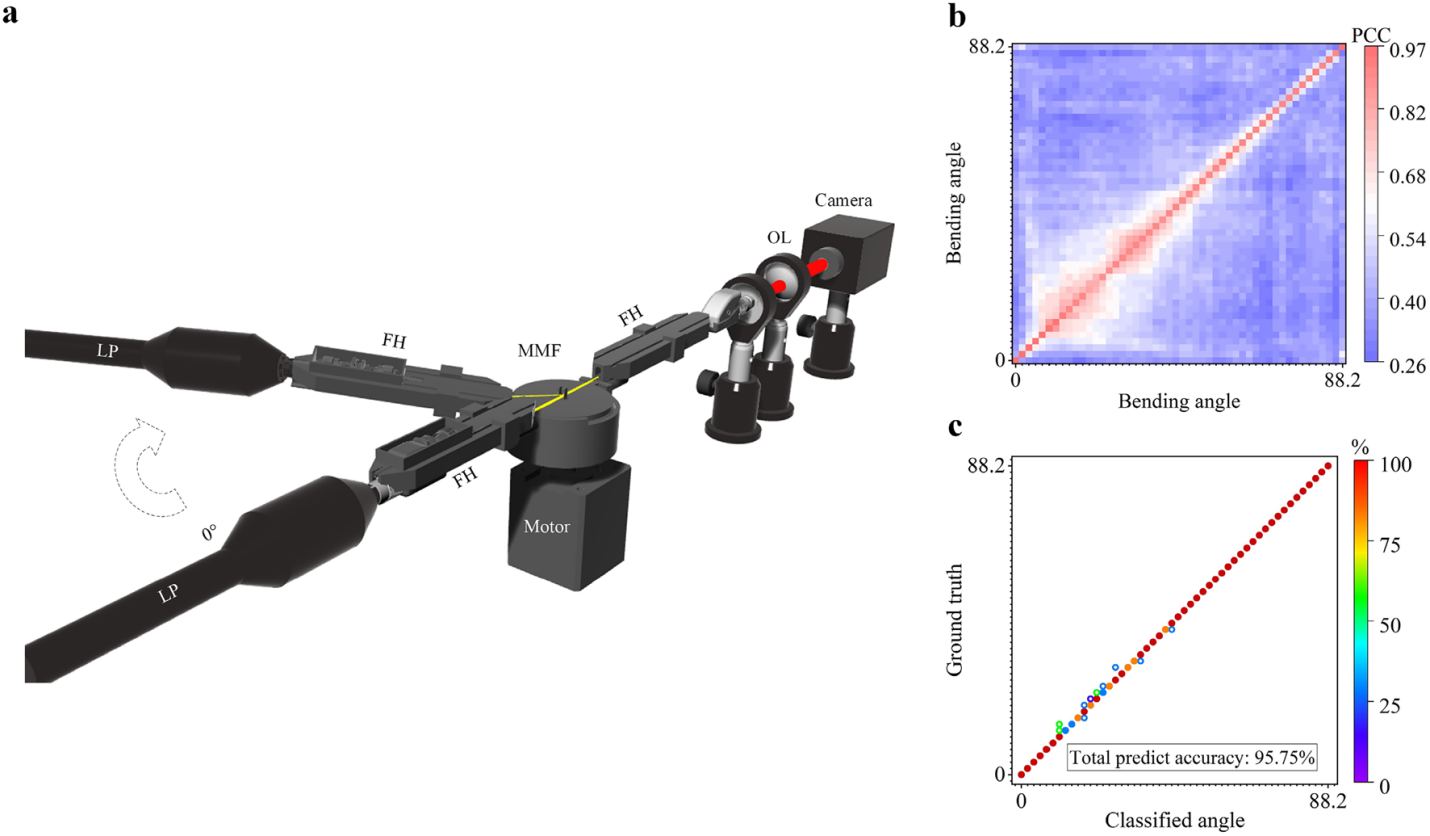
(**a**) Experimental setup for one-point motor bending angle classification. (**b**) Average PCC for one-point motor bending (the average PCC compared themselves with all pictures in other angles). (**c**) The KNN prediction result with 50% dataset as the training data. The colored circles in c indicate the location and percentage of classification errors while the solid circular dots on the diagonal show the classification accuracy.

We then set up an electrical-motor driven system to test the classification of different bending angles at one deformation location on a piece of MMF (Fig. 2a). A 20 cm long MMF and electric motors (RS PRO Hybrid Stepper Motor 0.9°, 0.44 Nm, 2.8 V, 1.68 A, 4 Wires) were used in this experiment. The testing MMFs were secured in place on the motor by homemade 3D-printed fiber holders. An Arduino system was used to control the movement of the motor and to trigger the camera to record a speckle in a synchronized manner. One MMF end connected to the camera was fixed, and the other MMF end was movable and was fuse-spliced to a single-mode fiber connected to a laser pointer. The motor bent the MMF at one point with bending angles ranging from 0° to 88.2° at a step of 1.8°. At each bending angle, the camera recorded one speckle image. We allowed 5 s for the system to fully settle before each image acquisition. By repeating 50 different angles bending for 16 times, the entire process took about 1 h and in total 800 datasets were collected (16 speckles for each bending angle). Figure 2b is the average PCC of speckles between bending angles. The correlation coefficient decreased to 0.3 after several times of deformation. It can be seen that the average PCC between speckles collected at the same bending angle is higher than those between speckles collected at different bending angles. Using the first recorded 400 data to train the KNN and the rest 400 as the test data, we were able to achieve a classification accuracy of 95.75% (Fig. 2c). The small classification errors are believed to be a result of the variability of the motorized-bending system, i.e. the MMF and the motor may have not returned to exactly the same state after each movement.

The results presented above suggest: (i) the deformation applied at different points along the same MMF results in different speckle patterns and the machine learning algorithms (i.e. KNN here) is able to distinguish such subtle differences; and (ii) different degrees of deformation at the same point also result in different output speckles, which can subsequently be classified by KNN. These forms a good basis of the following complex deformation sensing experiments.

### 2D multi-point deformation experiment

**Figure 3:**
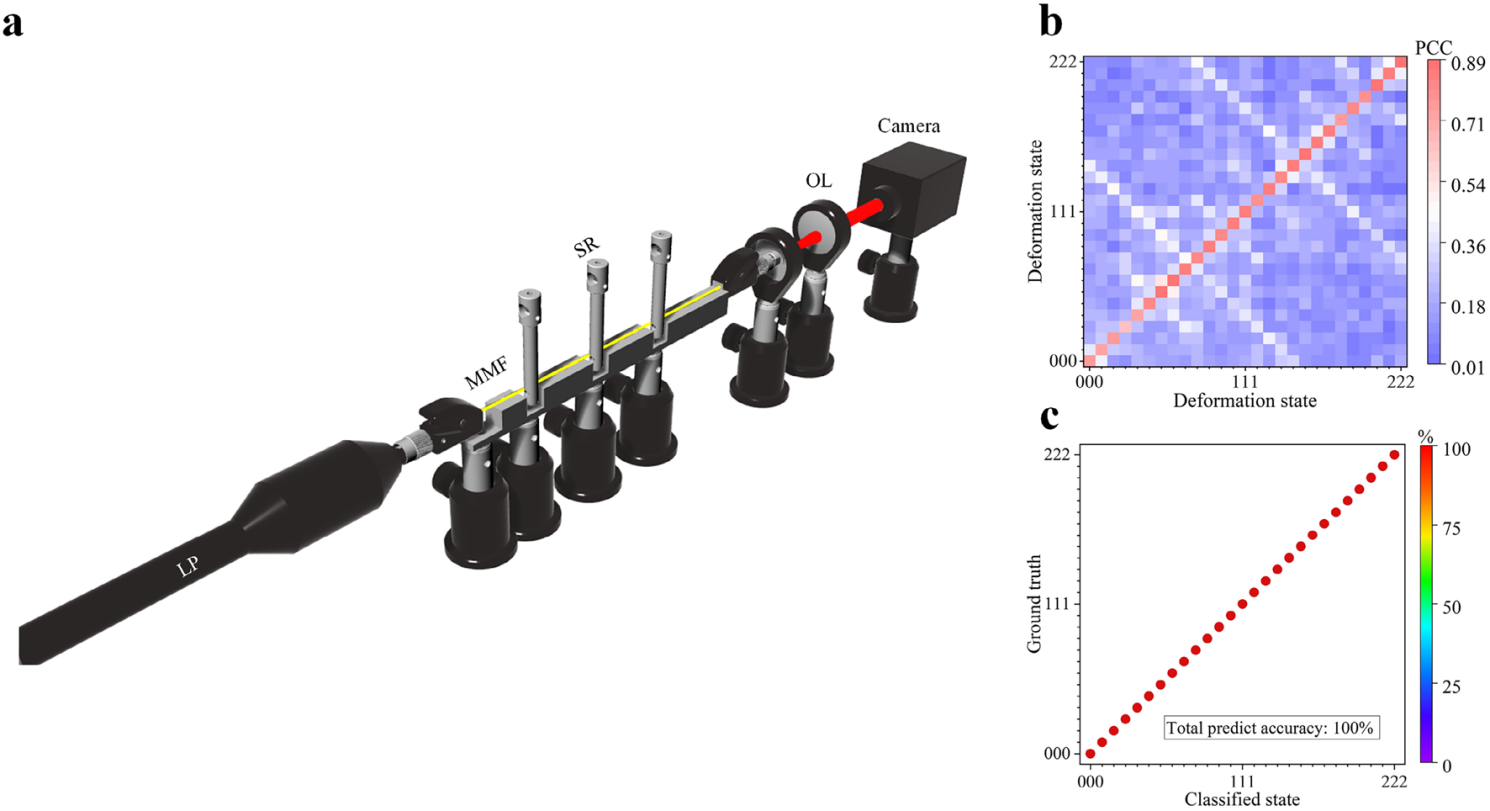
(**a**) Experimental setup for 2D multi-point deformation classification. (**b**) Average PCC between the speckle collected in the first measurements and speckles collected in the rest measurements. (**c**) Normalized confusion matrices for the KNN classification. The deformation states are defined by a deformation coordinates (*Y*_1_, *Y*_2_, *Y*_3_), where x of the *Yx* element represents the *x*-th bending rod, and the value of each element *Yx* defines the bending displacement step, i.e., 0 is 0 mm, 1 is 0.05 mm and 2 is 0.1 mm.

Next, we tested the performance of our sensor for two-dimensional (2D) multi-point bending. We used a 23 cm long MMF and fixed 12 cm section of the fiber as the experimental deformation region (Fig. 3a). A homemade fiber holder separated this bending region into 4 equal-length 30 mm long sections. Three 6 mm-diameter steel rods were used to apply displacements at the joint between these sections. For each step of the displacement, one steel rod was translated by 0.05 mm to deform the MMF, and for each steel rod there were three displacement values (i.e. 0 mm, 0.05 mm and 0.1 mm). Hence, there were 27 deformation states in total. Note that here we used a much smaller displacement (0.05 mm) compared with one-point experiment, to show the high sensitivity of the system. Similarly, before collecting a speckle we allowed 5 s for the system to settle after the displacement. The deformation process was repeated 50 times. In total, 50 speckles were collected for each deformation state and the entire process lasted about 2 h. Figure 3b shows the PCC between the states of the MMF recorded. The average PCC calculated for speckles of the same deformation state was the highest compared with the PCC between speckles from different deformation states. The correlation coefficient decreased to 0.2 after a few times of deformation. Using the first half recorded data to train the KNN and the rest as the test data, 100% classification accuracy was achieved as shown in Fig. 3c.

### 3D deformation experiment

**Figure 4:**
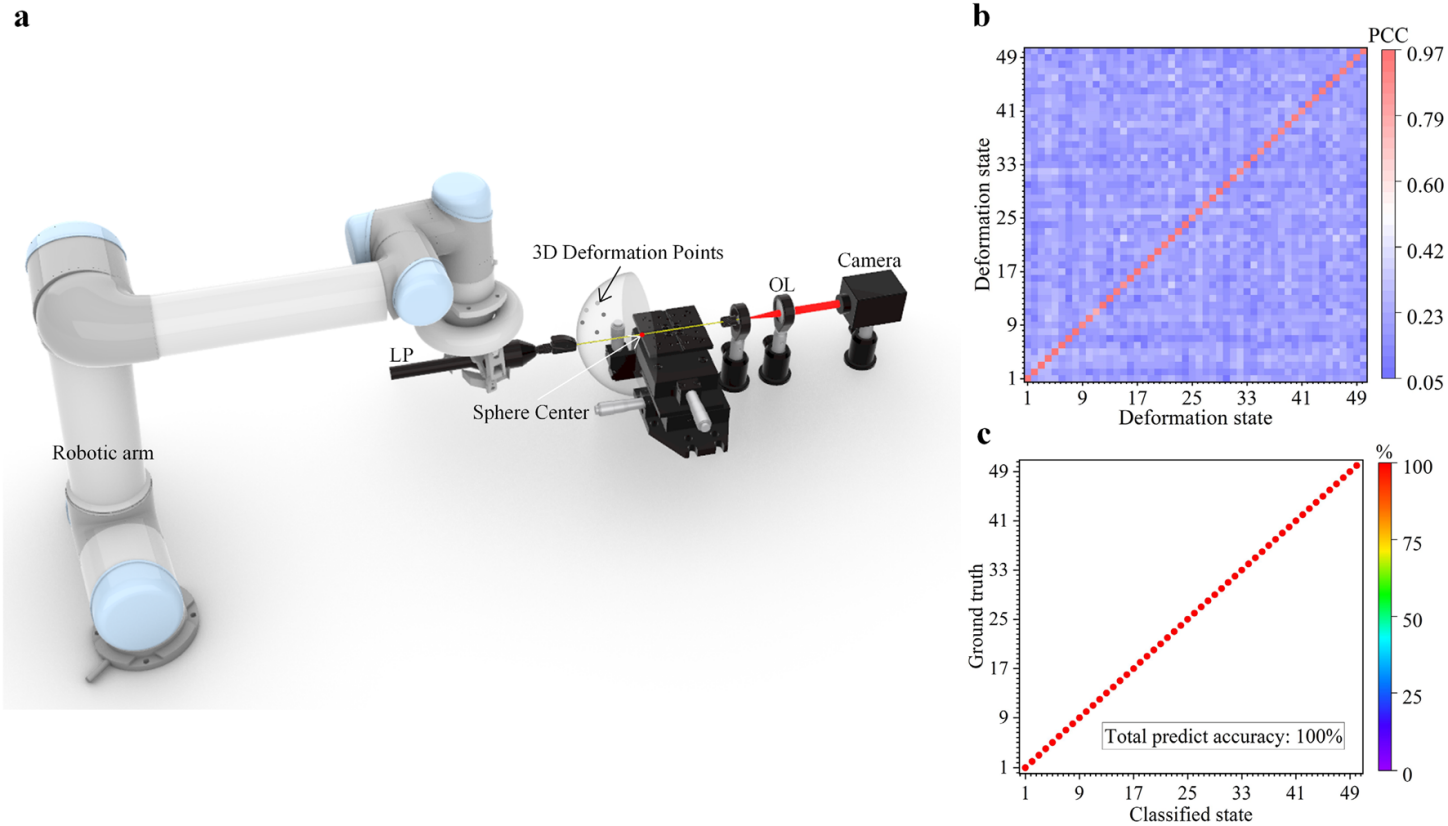
(**a**) Experimental setup for 3D one-point deformation classification. (**b**) Average PCC for 3D one-point bending between the speckles collected in the first measurement and speckles collected in the rest measurements. (**c**) The KNN classification result for 3D one-point bending using first half collected data as the training dataset and the second half data as the test dataset.

Finally, we attached our MMF sensor to a robotic arm (UR5, Universal Robot Company) for the proof-of-concept demonstration of three-dimensional (3D) deformation sensing. We first attached a 5 cm section of a 12 cm long MMF to a 5 cm-long end-effector section of the robotic arm for 3D one-point deformation test, as shown in Fig. 4a. The movement of the top section of the robotic arm follows a spherical Fibonacci lattice and the tip of the robotic arm can reach any points on a half spherical surface. The attached MMF therefore follows the same movement pattern as the robotic arm and is tested for 3D one-point bending. Based on the spherical Fibonacci lattice, we generated 50 different points on a 5 cm radius half sphere and fed the point coordinates to the end effector of the robotic arm. The laser pointer and the MMF were stabilized on the end effector, the MMF was deformed together with the robotic arm and followed the same 3D bending angle. The output speckle from the other end of the MMF was acquired by a camera. After the robotic arm reached a 3D deformation state, we allowed 3 s for the system to stabilize before the camera recorded a corresponding speckle. Note that we used a short waiting time (3 s) here instead of 5 s used in the previous experiments, as 3 s was found sufficient and additionally we can shorten the overall data acquisition time. These 50 3D deformation states were repeated 16 times within 1 h. The average PCC between speckles collected at the same bending state were much higher than the PCC between speckles collected at different bending states (Fig. 4b). The correlation coefficient decreased to 0.2 after several times of deformation. Again, we trained the KNN using first recorded 400 samples and validated the bending classification with the rest 400 data recorded later. Figure 4c shows the classification accuracy for 3D one-point deformation. These results indicate that all 3D one-point deformation of the fiber can be successfully classified, and a classification accuracy 100% can be achieved. In addition, we also achieved 100% classification accuracy for 3D one-point deformation of the MMF using the Robotic arm at the same bending degree, but different orientation, and the results are given in Supplementary Information 1.

**Figure 5:**
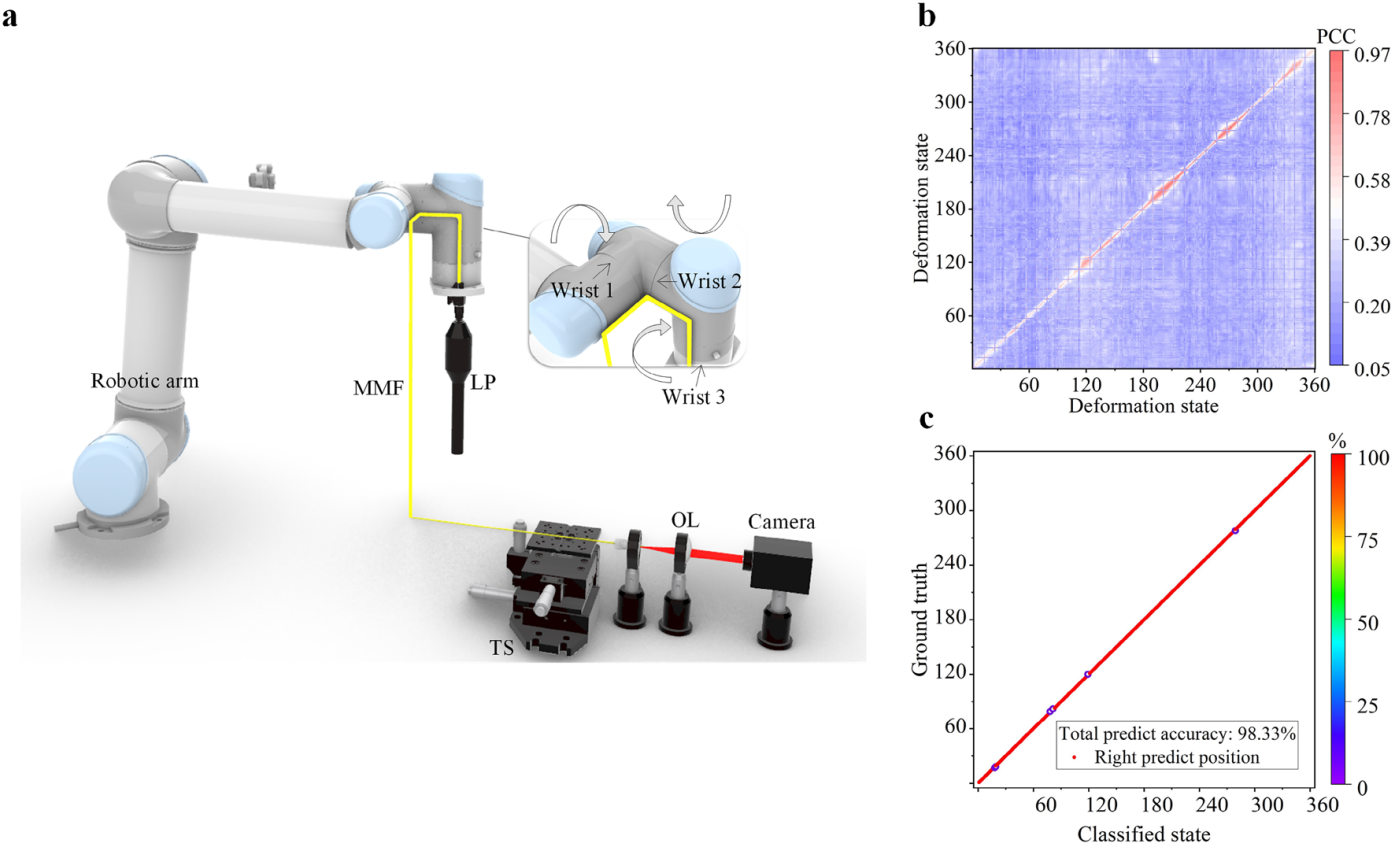
(**a**) Experimental setup for 3D three-point deformation classification; (**b**) Average PCC for 3D three-point bending between the speckles collected in the first measurement and speckles collected in the rest measurements. (**c**) KNN classification result using the last 20% recorded data in 3D three-point bending. The blue-colored circles in c indicate where the classification is unsuccessful.

Based on the 3D one-point deformation results above, we tested 3D three-point deformation sensing using a 220 cm-long MMF by attaching a 43 cm section of the fiber onto the entire robotic arm and covering three bendable joints, as shown in Fig. 5a. We used sealer tape to secure the fiber to the surface of the robotic arm (expect for the robotic arm bending joints, where the fiber was not covered by the sealer tape). The 3D three-point deformation was achieved by rotating these three robotic joints, resulting in the corresponding deformation on the attached MMF. For each bending joint, the bending angle can vary in the range from − 30° to + 30° with a minimal step of 1°. 360 deformation states in combination of movements of these three robotic joints were randomly generated and were repeated 5 times within 1 h. The camera recorded one speckle image within 1 s every time when the robotic arm moved to a bending state and a total of 1800 speckles are recorded. Similar to the electric motor experiment presented in the previous section, there appeared instabilities induced by relative fiber movements, i.e. the MMF may have not returned to the exactly same deformation state even though the robotic arm is back to the same state. This can be seen in the average PCC shown in Fig. 5b, where a reduced correlations between speckles collected at the same bending state are noted. The speckles have a low average correlation. We believe that such an instability is not the inherent property of the MMF sensor, but a result of the inevitable relative movements of the fiber under the sealer tape when the robotic arm was moved. Nevertheless, 98% classification accuracy was still achieved as shown in Fig. 5c when we used the first 80% recorded data to train the KNN and the rest 20% data as the test dataset. Further experimental results for 3D multi-point deformation are provided in Supplementary Information 2.

## Discussions and conclusion

One foreseeable challenge when applying the single MMF deformation sensor in real world is the high variability of the MMF output speckles, which is sensitive to the environment conditions, including temperature, vibration, and mechanical stress. Any small changes in these can lead to variations of the speckles, as shown in Supplementary Information 3 where the decorrelation of the output speckles along the time is clearly seen. The drifting of the MMF output speckle can be minimized by specially designed packaging and fixtures. To compensate the drifting, more training data can be included to enable the neural network to learn all possible variations^29^, or to update the neural network continuously by semi-supervised learning^31^.

Most experiment results presented in our work show high classification accuracy of 100% or very close to 100%. We believe that those observe classification errors (although only a few) are not the inherent error of the MMF bending sensor itself but are mainly due to the experimental setup, including the errors of the motor, the elasticity of the fiber jacket material after being stretched, and the small but undesirable relative movements between the taped-fixed fiber and the robotic arm in each robotic arm movement. Again, these can be improved with a specially designed packaging to secure the fiber on the structure or device to be monitored.

In summary, we have demonstrated 3D multi-points deformation sensing via a single MMF using machine-learning based speckle classification. The demonstrated single MMF deformation sensor is advantageous compared with traditional optical fiber deformation sensors in several aspects. Firstly, the transducer itself is a standard MMF and is therefore extremely low cost. The sensing system can be incorporated as a compact and wearable device where only a light source and an image sensor are needed, without requiring complex wavelength interrogation systems such as those needed for FBG sensors. Secondly, our single MMF deformation sensor can be applied to various types of deformation and a good resolution is achieved. As shown in our experiment, a 0.3 mm spatial resolution is achieved in Fig. 1, and a displacement as small as 0.05 mm can be successfully classified along the MMF (Fig. 3). Our experiment results prove that the single MMF speckle-based deformation sensor has good flexibility and sensitivity. Regardless of the influence of variations such as temperature and vibration, a resolution of 1° for 3D deformation is demonstrated in our experiment. It is possible to achieve even smaller resolution in micrometer scale (e.g. 5 µm) as demonstrated in Refs.^32–34^. Thirdly, 3D multi-point deformation is achieved via a single standard MMF and this is expected to be extended to more complex deformation or 3D shape sensing. In comparison, FBG based 3D shape sensing requires multiple fibers or specially designed multicore fibers. The advantages of our MMF deformation sensor include: high degrees of freedom, simple detection setup without requiring additional wavelength interrogation devices, and an overall low-cost system. This single MMF based 3D multi-points deformation sensing technique may find applications in limb motions monitoring, surgical tools manipulation, wearable device sensing and robotic control.

## Methods

### Optical sensing experimental setup

Output from a laser pointer (650 nm, 1 mW, KLS-006, Kelushi) was coupled into MMFs (50 μm-core, 0.22NA, FG050UGA, Thorlabs) of different lengths between 110 and 2200 mm according to the requirements of different bending experiments). It has been experimentally demonstrated that multimode fibers with larger cores or smaller numerical apertures are more sensitive to deformation^35^. A longer MMF increases the variations between speckles for the same deformation but at the same time introduces more instability. The other end of the MMF was imaged with a digital CMOS camera (2048 × 2048 pixels, 6.5 μm × 6.5 μm pixel sizes, C11440-22CU, Flash4.0, Hamamatsu) through a 30 mm focal lens. All fibers were bare fibers and were tested without protective jacket or connectors. How the optical fiber speckle changes with deformation is given in Supplementary Information 4.

### Optical sensing experimental setup

The KNN algorithm was used to analyze the experimental data owing to its simple implementation and good classification performance^36–38^. KNN is simple and requires tuning only one hyperparameter (the value of *k*), while neural network training involves many hyperparameters controlling the size and structure of the network as well as the optimization procedure. For advanced deep neural networks such as convolutional neural network (CNN), they require a much larger size dataset. Due to the small dataset size in our experiment, KNN is ideally positioned to classify the speckles. Another important justification is that compared it with deep neural networks, such as CNN, KNN can obtained a similar accuracy in our experiment dataset. The results are provided in Supplementary Information 5. For more complicated deformations, deep neural networks could be used for better recovery, for example, to predict states not included in the training.

The KNN classification is performed by using a training dataset which contains both the input and the target variables and then by comparing the test data which contains only the input variables to that reference set the distance of the unknown to *k* nearest neighbors determines its class assignment by either averaging the class numbers of the *k* nearest reference points or by obtaining a majority vote for them.

The speckles were firstly normalized and standardized before applying them with the KNN. The size of the speckle was set to 120 × 120. Image preprocessing was done by using *scaler.fit_transform* function within Python *sklearn.preprocessing* Library. Bending state labels were inserted in each figure and at the same time corresponding speckle patterns of different bending states were identified. Subsequently, the labels and their corresponding speckles were set up in a dataset. This dataset was then split into two data groups for training and testing, respectively. After the training of KNN, speckle images in the test dataset were sent to KNN to obtain the predicted bending-state labels. The predicted labels were compared with the original bending state labels to determine the accuracy of KNN classification.

The kNN algorithm involves three main factors: the training set, the distance measurement, and the size of *k*. The best model hyperparameters is *k* equals to 3. The distance metric for the proximity evaluation in this paper was chosen to be the Euclidean distance.

## Supplementary Information

Supplementary Information.

## Data Availability

The datasets generated and/or analyzed during the current study are available from the corresponding author on reasonable request.
