# Supplementary material for: Learning to sense three-dimensional shape deformation of a single multimode fiber
Source: Sci Rep. 2022 Jul 25;12:12684. doi: 10.1038/s41598-022-15781-8 (PMC9314325; doi:10.1038/s41598-022-15781-8)
Supplement: Supplementary file 1 — Supplementary Information. [file 41598_2022_15781_MOESM1_ESM.docx]

Learning to sense three-dimensional shape deformation of a single multimode fiber: Supplementary Information

Xuechun Wang^1^, Yufei Wang^1^, Ketao Zhang^1^, Kaspar Althoefer^1^, and Lei Su^1,*^

^1^School of Engineering and Materials Science, Queen Mary University of London, London, E1 4NS, United Kingdom.
^*^corresponding.author: [l.su@qmul.ac.uk](mailto:*l.su@qmul.ac.uk)

1. 3D one-point robotic arm deformation at the same degree of bending angles but different orientations


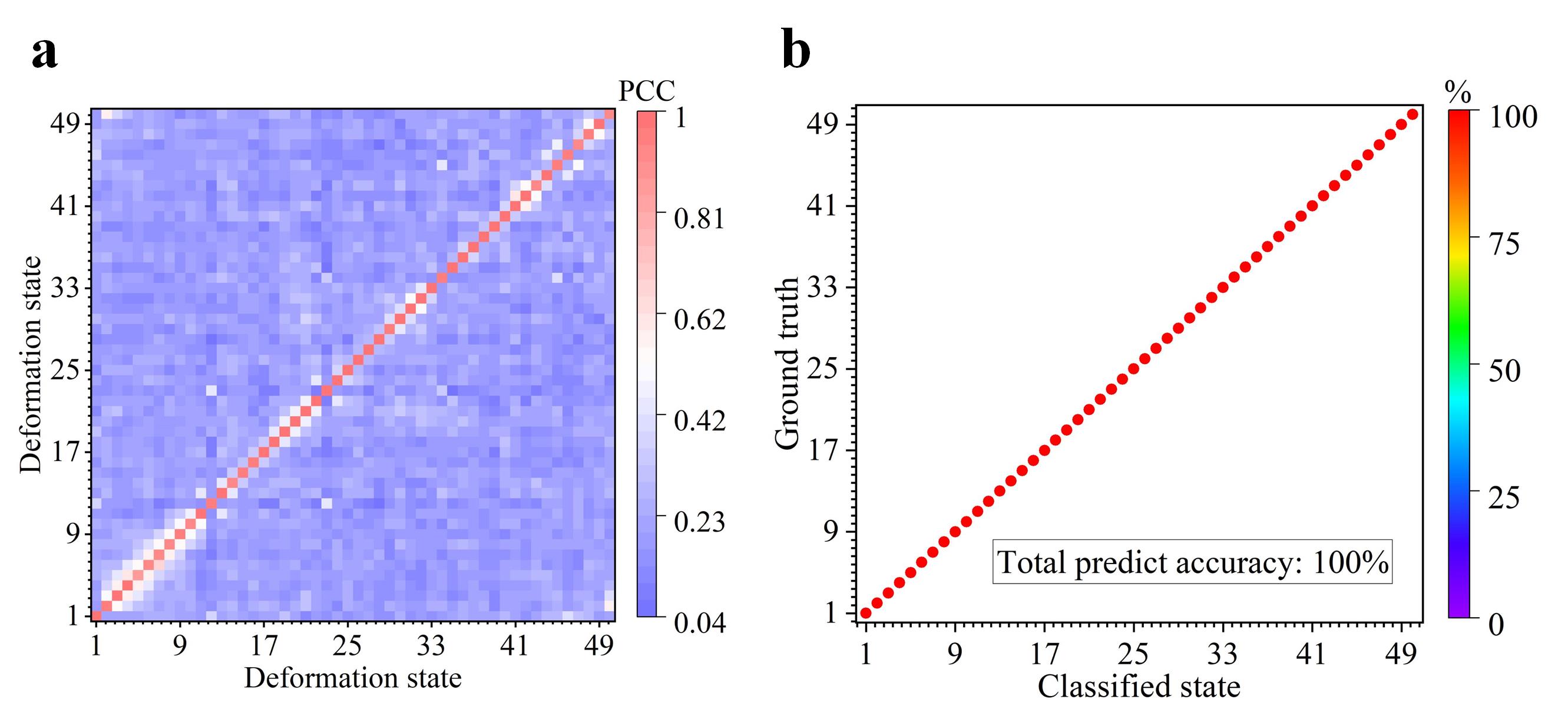


**Fig. S1.** a. Average PCC for 3D one-point same degree but different orientation bending (calculated between the first measurements and the rest measurements) b. kNN classification result using the first recorded 50% data to train the kNN and the rest 50% recorded data as test dataset.

In the configuration shown in Fig 4a in the main text, we generated 50 points at one latitude on a sphere. These 50 points were 45 degrees to the center of the circle. We used a 5-cm-long end-effector section of the robotic arm to rotate a 5 cm section of a 12-cm-long MMF. We allowed 3 seconds for the system to stabilize. At each bending orientation, the camera recorded one speckle image. The bending process was repeated 16 times. A total of 400 pictures were collected within 1 hour. As shown in Fig. S1a and S1b, the average PCC between speckles collected at the same bending angle is higher than those between speckles collected at different bending angles. A classification accuracy of 100% is achieved.

2. 3D three-point deformation experiment using the robotic arm


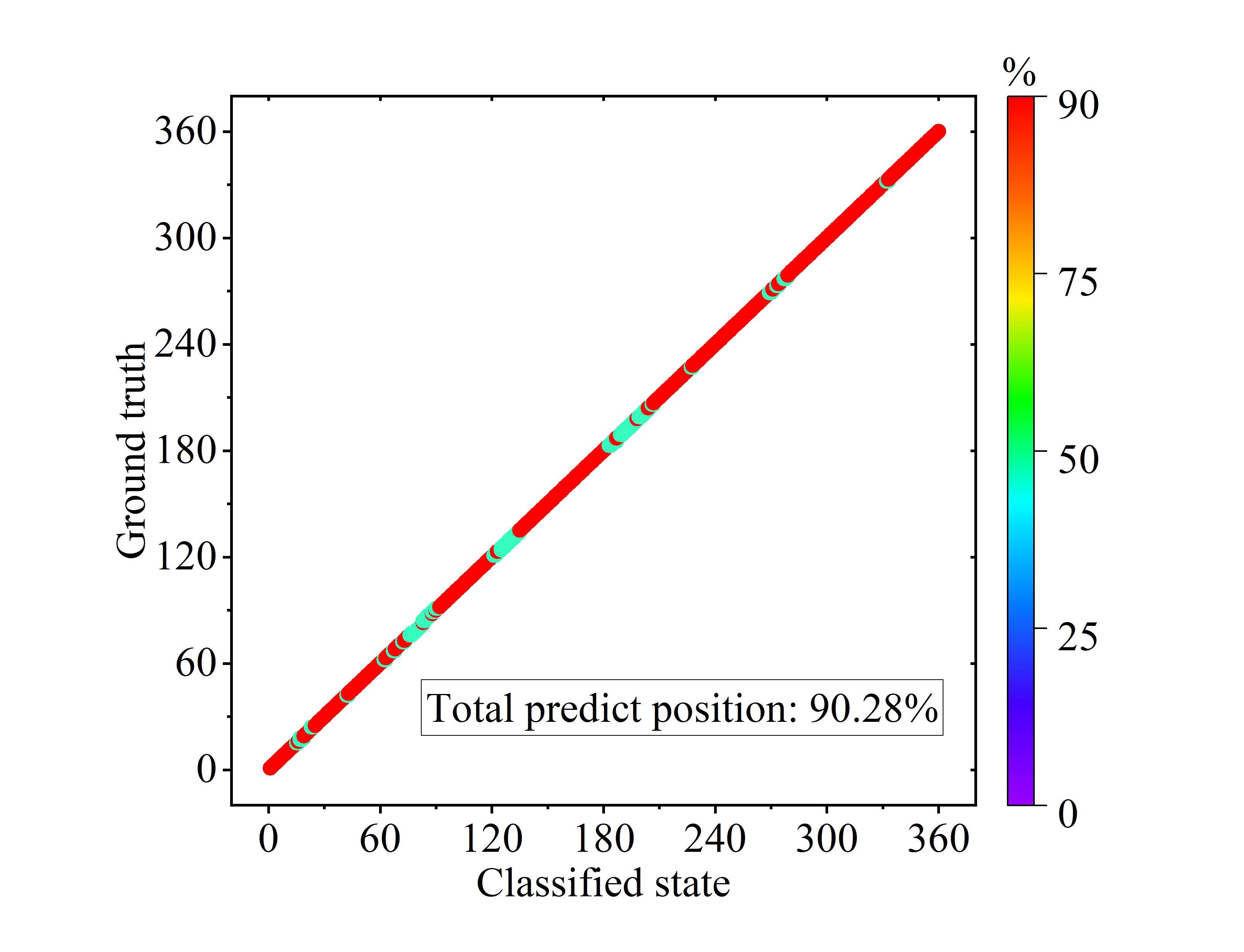


**Fig. S2.** The same dataset as that used in Fig. 5 in the main text. Here, the kNN classification results were obtained by using the first recorded 60% data to train the kNN and the rest 40% recorded data as the test dataset.

The 3D 3-point deformation state was set as follows: The range of motion for each wrist bending is 60°. The deformation states were defined by bending coordinates (*Y_1_*, *Y_2_*, *Y_3_*), where *x* of the *Yx* element represents the *x*-th bending wrist, and the value of each element *Yx* defines the bending angle step. Taking the initial state as (-30, -30, -30), we bent the first wrist range from -30° to 0° (0, -30, -30) and bent the second and third wrist with the same ranges, respectively. Next, the first wrist was bent to 30° (30, 0, 0) followed by the second and third wrist bending. Then the first wrist was bent to 0° again (0, 30, 30) and the second and third wrist were bent in the same way. We bent the first wrist to its initial angle (-30, 0, 0) and the second and third wrist followed again. Before we start the next loop, we bent the last wrist for one degree and bent back in order to get 360 different bending state in total.

3. Stability


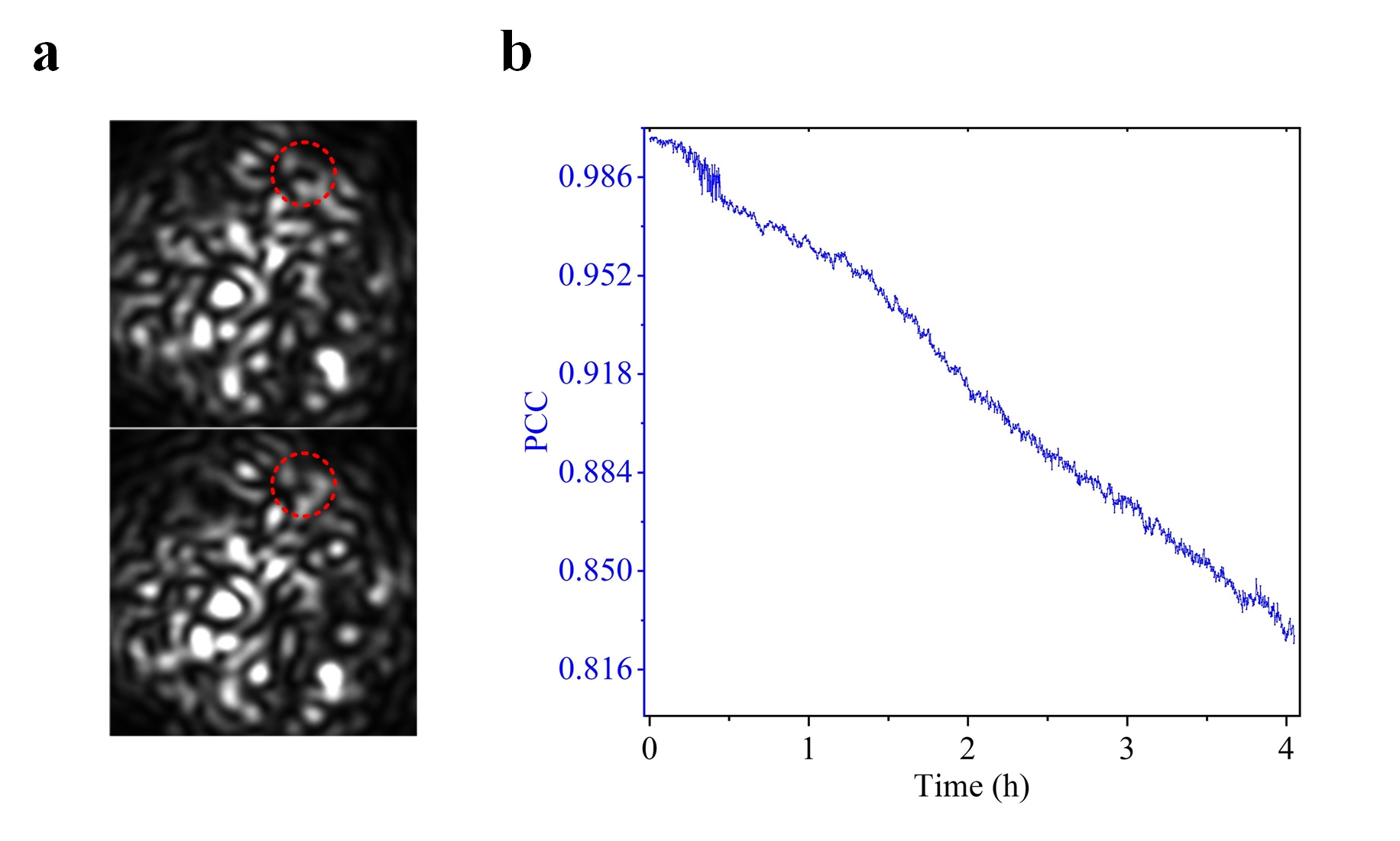


**Fig.S3.** Stability of the MMF output speckle over the time. a. The first and last speckle within 4 hours b. PCC changes within 4 hours.

We tested the stability of a 12-cm-long MMF by capturing one output speckle every 10 s within 4 hours. Fig. S3a shows the first and last speckle pattern changes with time. The two speckles are slightly different to the naked eye. The slight changes are believed to be a result of the variability of the environment. Fig. S3b shows the PCC comparing speckles with respect to the first speckle. The speckle correlation coefficient can still achieve 0.83 after 4 hours.

4. Speckle changes


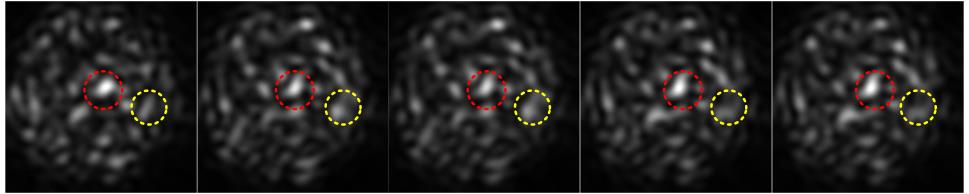


**Fig. S4.** Output speckles corresponding at different deformation states

Fig. S4 shows the speckle changes when the robotic arm was rotated 0.5° per step along the axial direction of the fixed MMF. The speckle marked with red circle start from a bright dot to a long strip. The intensity was increasing gradually. The speckle area within yellow dashed area begins with a long strip to a dot and changed to a rectangle. Although speckles appear to follow no discernible cycle, it was changing gradually. In practical, the speckle changes can be regarded as a continuous process if large number of speckles were captured. Accordingly, more powerful deep neural networks, such as convolutional neural network (CNN), will be required to predict the probability of the corresponding deformation state without the need to train each state. Fig. S5 shows some speckle examples in multi-point 2D deformation experiment.


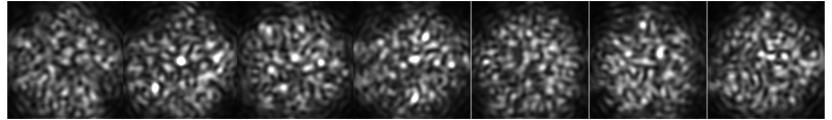


**Fig. S5.** Examples of speckle in multi-point 2D deformation

5. CNN performance on our dataset


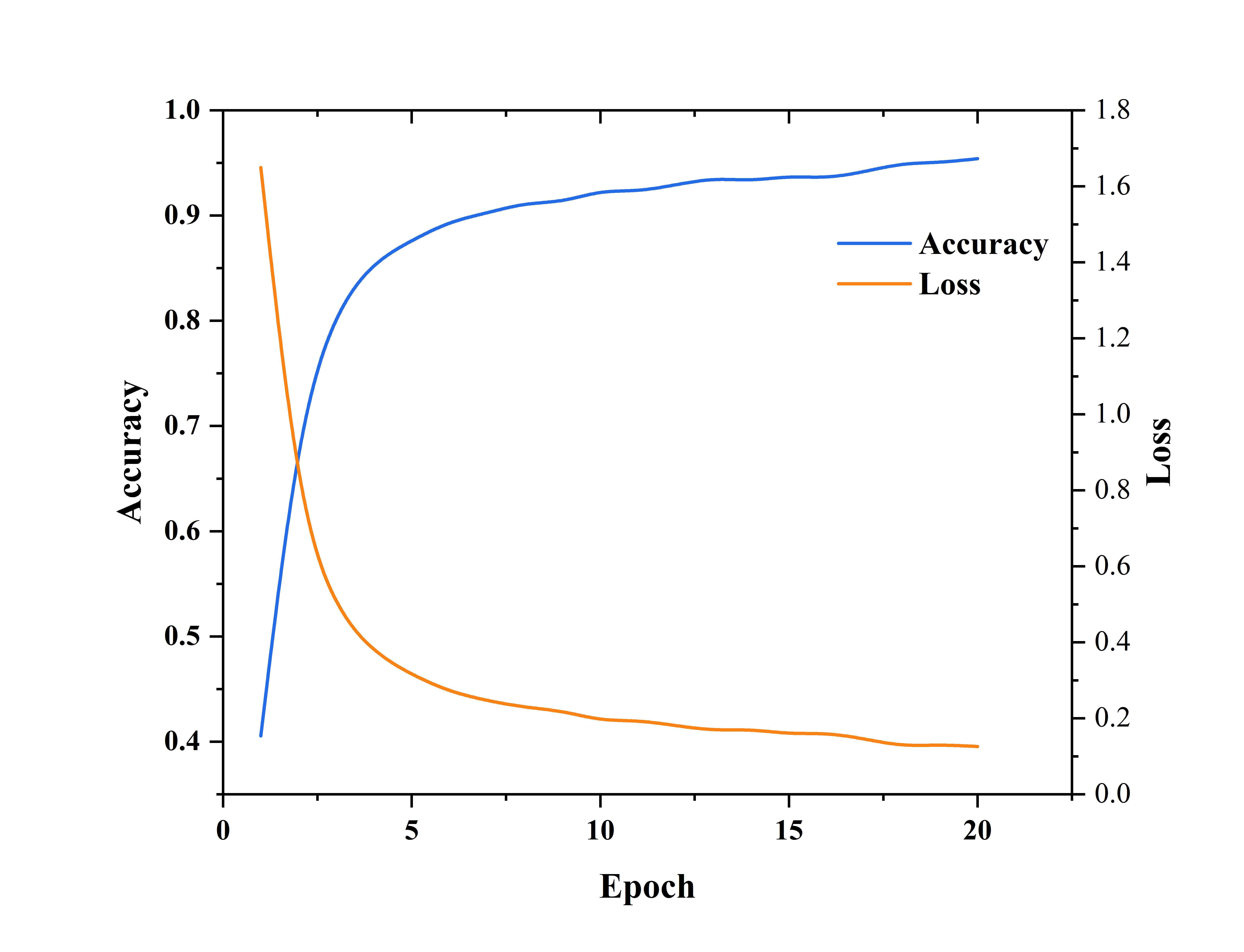


Fig S5. CNN training accuracy and loss based on 25 classes speckles

To demonstrate the effectiveness of CNN on our experiment, we tested one-point deformation using robotic arm. We allow 1 second for the system to stabilize before the camera recorded a corresponding speckle. The robotic arm moved with a step of 1°. A total of 25000 speckles were recorded. The total dataset is 1000 for each state. Visual Geometry Group 16 (VGG16) is a CNN model which contains 13 convolutional layers, three fully connected layers and five pooling layers. Experiments performed on VGG16 for image datasets are trained on the 80% training set images and tested on the 20% testing set. For the experiment results, we set the parameters for CNNs. The learning rate is 0.0001, Batch-size is 50, epoch is set to 20 and the ratio of dropout for VGG-16 is 0.5. According to Fig. S5, we were able to achieve a classification accuracy of 95.75%. For the same dataset setting, 91.02% classification accuracy was achieved for *k*NN. Table S1 shows the model evaluation times.

Table S1. Mean evaluation time of two models on one test data.

| Model | Network output runtime (second) |
| --- | --- |
| *k*NN | 0.044 |
| CNN | 0.001 |
